# Disaster safety assessment of primary healthcare facilities: a cross-sectional study in Kurdistan province of Iran

**DOI:** 10.1186/s12873-021-00417-3

**Published:** 2021-02-23

**Authors:** Arezoo Yari, Yadolah Zarezadeh, Farin Fatemi, Ali Ardalan, Siamak Vahedi, Homa Yousefi-Khoshsabeghe, Mohsen Soufi Boubakran, Farzam Bidarpoor, Mohamad Esmaeil Motlagh

**Affiliations:** 1 Social Determinants of Health Research Center, Research Institute for Health Development, Kurdistan University of Medical Sciences, Sanandaj, Iran; 2 Research center for health sciences and technologies, Semnan University of Medical Sciences, Semnan, Iran; 3 Department of Health in Emergencies and Disasters, School of Public Health, Tehran University of Medical Sciences, Tehran, Iran; 4 Department of Cardiology, Faculty of Medicine, Kurdistan University of Medical Sciences, Sanandaj, Iran; 5 Department of Mechanical Engineering, Urmia University, Urmia, Iran; 6 Department of Pediatrics, Ahvaz Jundishapur University of Medical Sciences, Ahvaz, Iran

**Keywords:** Primary healthcare facilities, Risk assessment, Safety assessment

## Abstract

**Background:**

Kurdistan province of Iran is among disaster prone areas of the country. The Primary Health Care facilities in Iran deliver health services at all levels nationwide. Resiliency and flexibility of such facilities is important when a disaster occurs. Thus, evaluating functional, structural, and non-structural aspects of safety of such facilities is essential.

**Methods:**

In this cross-sectional study, the instrument used to evaluate four sections of functional, structural, non-structural, and total safety of 805 healthcare facilities in Kurdistan Province was the safety evaluation checklist of primary healthcare centers, provided by the Iranian Ministry of Health and Medical Education. Each section scored from 0 to 100 points, and each section of the safety was classified to three safety classes according to their total score: low (≤34.0), average (34.01–66.0) and high (> 66.0).

**Results:**

The levels of functional, structural, non-structural and total safety were equal to 23.8, 20.2, 42.3 and 28.7, out of 100, respectively. Regarding the functional safety, rapid response team scored the highest, while financial affairs scored the lowest. Nevertheless, in structural and non-structural sections, the scores of different items were almost similar.

**Conclusions:**

The results of the study revealed that safety score of primary healthcare facilities in general was unsatisfactory. Thus, promoting preparedness, resilience and continuity of service delivery of these facilities are essential to response to disasters and emergencies. The finding of this study could be beneficial for national and provincial decision-makers and policymakers in this regard.

**Supplementary Information:**

The online version contains supplementary material available at 10.1186/s12873-021-00417-3.

## Background

World Health Organization (WHO) considers public health as a set of organized actions which attempt to prevent disease, improve health, and increase the longevity of population [1]. The focus of Primary Health Care (PHC) is on delivering essential services to improve the health condition, which results in resiliency for society, and consequently emergencies can be dealt with efficiently [2]. The occurrence of disasters in societies causes serious damage and influences them severely. From the date Hyogo Framework for Action was approved to 2015, it was estimated that thousands of people had lost their lives and millions had become homeless due to disasters globally [3]. Natural disasters, emergencies, and other crises have a direct effect on people and society’s health and influence it through causing trouble for health systems, equipment, and services [2].

Disaster risk management prevents or reduces the rate of deaths, accidents, diseases, disabilities and mental problems [2]. Therefore, policies and strategies should focus on equipping and preparing PHC facilities because they can reduce the vulnerability of families, societies, and public health systems, caused by disasters and emergencies [2]. Continuous training and exercises as strategies for improving functional safety can improve preparedness and resiliency of health staff and people against disasters and emergencies [4]. According to WHO, lack of training to prepare for disasters on national, and community levels has been one of the main reasons for high casualties and damage from disasters [5]. Additionally, availability and continuity of public health services to all populations are one of the principal actions of public health in order to reduce disaster risks [1, 6]. Countries are encouraged to improve health systems along with international commitments in order to improve preparedness for disasters. Regarding global significance of the issue, the World Health Assembly of WHO approved a resolution on strengthening national health emergency and disaster management capacities and resiliency of health systems in May 2011 [7].

The United Nations post-2015 framework for disaster risk reduction announced the aims of negotiations on disaster risk reduction as follows: increase in health system flexibility, incorporation of disaster risk reduction into healthcare programs, and capacity building especially at local level [6]. One of the expected outcomes from Sendai framework in addition to reducing casualties due to disasters is to lessen the damage to basic infrastructures and service-delivery facilities [3]. Some Studies indicate that the main reason for most of the damages in the health facilities is related to inappropriate site selection for the building, lack of proper design or insufficient maintenance [8]. In the earthquake of the Iranian city of Bam in 2003, more than 90% of health facilities were demolished [9]. Further, after the 2004 Indian Ocean tsunami, in Sri Lanka at least 92% of the health physical infrastructure were partially or fully damaged [10]. Indeed, a combination of the structural and non-structural safety and a high level of functional safety is required to ensure that PHC facilities are resilient enough to disasters and emergencies. If the safety in the mentioned domains increases, the flexibility of PHC facilities also increases [11]. Since Iran is a disaster-prone country, one of the public health concerns in the country is related to the harmful consequences of disasters [12]. Notably, these PHC facilities are the first level of contact between families and the health system in the governmental sector of Iranian health system [13].

The structure of PHC system was established in Iran in 1985. In Iranian health system, each health house provides healthcare services to about 1200 inhabitants of each village or some villages by trained healthcare workers called Behvarz. In more populated villages, there are rural PHC facilities staffed by a physician and a team of up to 10 health workers providing healthcare for more complex health services such as child and mother care, reproductive health, environmental health and mental health. This service is provided by the government along with health houses [13].

Each rural PHC center covers almost 7000 inhabitants. In urban areas, PHC facilities provide similar health services as health houses and rural PHC facilities. This network is managed by district PHC facilities, under the supervision of Medical Sciences Universities. Economic issues, village or city location, road damages, and affordability of the service cost in rural and urban areas are reasons why the majority of the population can or cannot access PHC facilities particularly after disaster occurrence [13, 14].

Totally, these 24,000 PHC centers across the country have been accounted as a good potential to deliver multi-health services in four phases (prevention and mitigation, preparedness, response, and recovery) of disasters to the population [12]. Therefore, the stability and safety of PHC facilities as well as trained staff are necessary for continuing the health care service delivery to affected people at time of disasters and emergencies [15]. The focus of this study, Kurdistan Province situated in the west of Iran, has an area of 28,235 km^2^ accounting for about 1.7% of the country’s area [16]. With regard to topographical, diversity of geographical and ethnicity, Kurdistan is one of the provinces prone to various disasters such as earthquake, floods, fires (especially on mountain forests), terrorist attacks, war, avalanche, blizzard, drought, and other risks. Different ethnicities seem to be different in terms of the availability of economic resources, education and culture of safety and preparedness for disasters. This impacts the knowledge، attitude and prevailing tendency to participate in actions to mitigate the effect of environmental disaster [17]. Furthermore, the Zagros fold-thrust belt crosses over this province and large earthquakes are expected to occur due to this fault in the province. Kurdistan Province is divisible into eastern and western areas in terms of seismicity, with more than 60% of the western area in this province including the cities of Kamyaran, Sanandaj, Marivan, and Baneh located in the high-risk zone [16]. Regarding disaster-proneness of this province and the low socio-economic indices, significance of the PHC centers stability and continuity in delivering health services is vital for affected people, particularly after the disaster occurrence. It is required to mention that a comprehensive safety assessment has not been conducted in all PHC facilities across the province. Therefore, it is crucial to collect data and provide precise information for health officials and decision makers in order to recognize the weak points of PHC facilities against disasters and emergencies so that their preparedness is improved. The aim of this study was assessing the structural, non-structural, functional and total safety and relevant risks for disasters in 805 primary PHC facilities at provincial, regional and local levels in Kurdistan province, Iran.

## Methods

### Design and setting

**Fig. 1:**
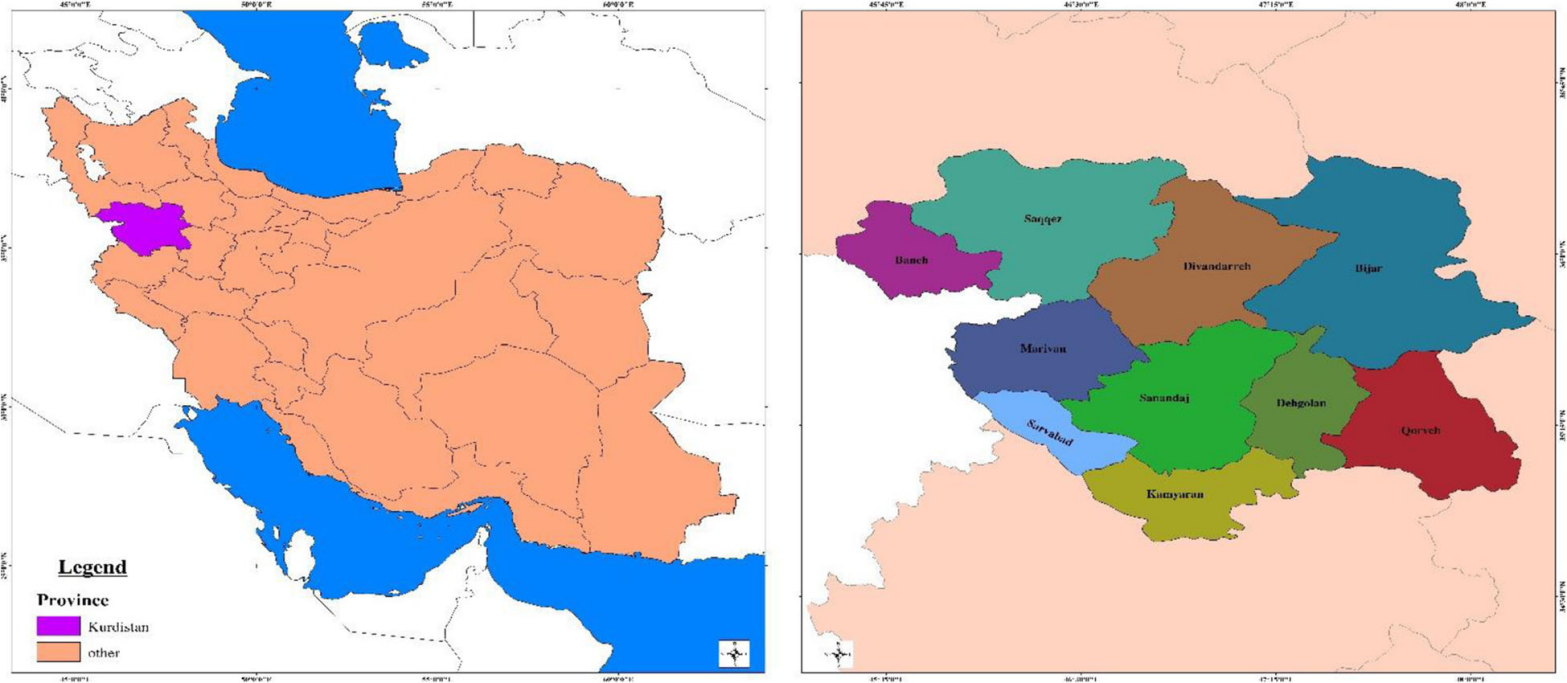
Kurdistan Province in Iran and 10 County of Kurdistan Province. These maps were constructed by authors using existing data in https://en.wikipedia.org/wiki/Kurdistan_Province at 02 Aug 2020.

This cross-sectional study was conducted at healthcare facilities in Kurdistan Province located in the west of Iran accommodating 10 towns in 2018 [18] (Fig. 1). The sampling method was census and 805 existing healthcare facilities were included in this study.

### Collecting data instrument

The checklist used in this study was developed based on Hospital Safety Index (HSI) instrument. World Health Organization (WHO) has established the HSI, which is a validated, international, multi-risk assessment instrument [19]. The checklist was first introduced for hospital safety assessment by PAHO in 2008 [20]. To translate and adopt HSI in Iran, in the first step, a multidisciplinary group of experts translated the checklist into Farsi. Then, four professionals in related fields of medical sciences and engineering reviewed the Persian version of the checklist in terms of application and appropriateness indicators. They also checked the accuracy of the translation. Then the compiled version was piloted in one of the hospitals in Tehran, capital of Iran. Also, content validity, content and weighting of indicators were assessed in the panel of experts and the face validity was assessed using views of the hospital personnel [21, 22]. moreover, its reliability was found to be acceptable with the score of 0.82 using Cronbach’s alpha coefficient [23].

The Iranian version of HSI was similar in structure of the tool and number of items to the original HIS version [21]. HSI has already been adopted for applying in the healthcare facilities with the purpose of safety assessment and was confirmed by other specialists as the collection data instrument [24]. HSI has also been used to assess the disaster safety in primary healthcare facilities around the world [25, 26].

In addition; We assessed the suitability of the checklist by an expert panel. Members of the panel were defined as individuals who were health in disasters and emergencies professionals with academic backgrounds working in primary healthcare system. We asked the experts to answer some questions regarding the suitability of the checklist for assessing healthcare facilities as well. The professionals affirmed the suitability of the checklist for assessing the healthcare facilities. This checklist has the capacity for measuring the disaster safety assessment in healthcare facilities via recognizing threatening hazards, assessing the functional preparedness as well as structural, non-structural and total safety assessment. The checklist does not ask any question requiring confidential answers. In case of missing data one of the members of the research team contacted the person, who had completed the checklist in the relevant facility.

The section of hazard recognition included 51 questions in five sub-sections including geological, climatic, social, biological, technological and man-made hazards. Also, the probability of risk occurrence was categorized to four levels: improbable, low, moderate and high. We assigned scores of 0 or < 1, 1, 2 and 3 to each category, respectively.

The section of functional preparedness included 241 questions in 34 sub-sections. Some of these sub-sections were as follows: organization and structure, preparedness programs, risk assessment, insurance, risk reduction measures, firefighting, and exercise. Preparedness level was categorized to three levels: acceptable, moderate and unacceptable. We assigned scores of 3, 2 and 1 to each category, respectively.

The section of non-structural safety included two sub-sections: general with 43 questions and technical with 110 questions. In the general sub-section, the safety of general equipment found typically in most offices is measured, while in the technical sub-section, the safety of specialized equipment of health facilities was measured. The safety level of non-structural components was categorized to three levels: safety rules have not been observed (low safety), safety rules have been partially observed (moderate safety), and safety rules have been fully observed (high safety). We assigned scores of 0, 1 and 2 to each category, respectively.

The section of structural safety includes five questions and scoring of this section is as the same as non-structural section.

In each primary healthcare facility, one health staff was assigned for the assessment coordination, data collection and data entry and reporting to research team. This person was usually the health in disaster management expert or a person in charge of health who was also responsible for providing health management in disasters or emergencies. These health staff completed basic disaster risk management courses and were responsible for health management in disasters and emergencies at their primary health care facility. These health staff, at different levels of health system had been trained about safety, risk and disaster concepts, data collection methods including field investigation, observation and interview, and scoring of checklists during two courses. Each course was organized in 3 days. The participants were monitored and evaluated at the end of second course by research team. During the survey period, two members of the research team were available during working hours to answer queries from the data collection team. The data collection process started simultaneously in all primary healthcare facilities in Kurdistan province from May 2018 and finished in August 2018.

### Data analysis

The completed checklists of each facilities were entered to the Excel software. The score of each section was calculated from 0 to 100scale and in order to homogenize the results, the score of all questions was considered from 0 to 100.Total safety score was the average scores of assessed sections in functional, structural, and non-structural areas calculated from 0 to 100. each section of the safety was classified to three safety classes according to their total score: low (≤34.0), average (34.01–66.0) and high (> 66.0).

## Results

**Fig. 2:**
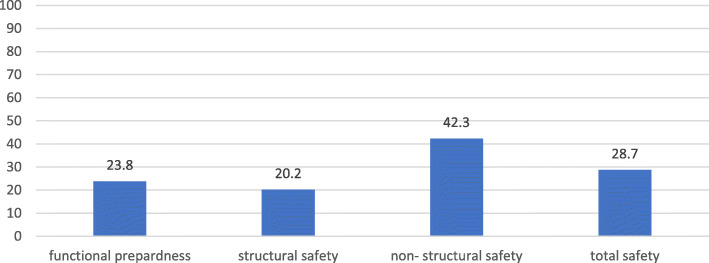
Safety Indicators in Primary Healthcare Facilities (%), Kurdistan, Iran

**Fig. 3:**
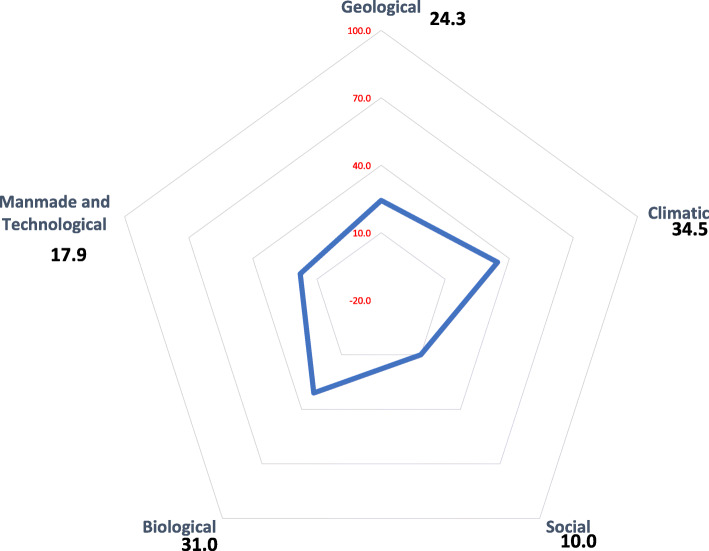
Threatening Hazard Type in Primary Healthcare Facilities (%), Kurdistan, Iran

According to the results of this study, the total safety score of primary healthcare facilities under assessment was equal to 28.7 in Kurdistan Province. The highest safety score was related to the non-structural section, while the lowest ones belonged to structural safety section (Fig. 2). In the section of hazard recognition, the most threatening hazard type of healthcare facilities throughout the province were related to climatic, biological, geological hazards with 34.5, 31, and 24.3% respectively (Fig. 3).

**Fig. 4:**
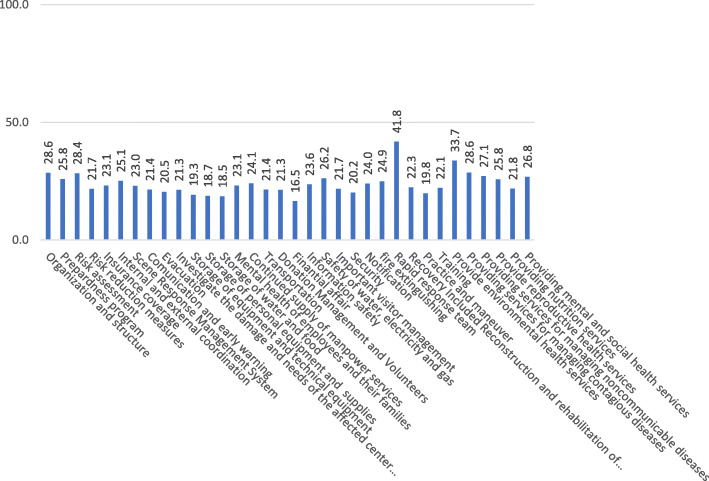
Functional Preparation Scales in Primary Healthcare Facilities (%), Kurdistan, Iran

The average score of assessing functional preparedness in all assessed healthcare facilities was equal to 23.8. The highest score of functional preparedness items were related to organizing rapid response team (41.8) and also, environmental health services delivery (33.7). The lowest score of functional preparedness items were related to financial affairs, water and food supplies, and providing appropriate Personal Protective Equipment (PPE) for staff with the score of 16.5, 18.5, and 18.7, respectively (Fig. 4). According to the type of healthcare facilities, the highest rate of  total safety belonged to the district health network (34.8). Also, the urban health centers obtained the lowest score in this section of assessment (25.8) (Table 1).

**Table 1:** Functional Preparedness, Structural, Non-Structural and Total Safety Score (%) according to Healthcare Facilities Type, Kurdistan, Iran

Type of Healthcare facility	Number	Functional Preparedness	Structural safety	Non-structural safety	Total safety
District Health Network	1	2.4	30	72.3	34.8
District Health Centers	10	41.7	33.6	27.3	34.2
Urban Health Centers	33	14.8	17.4	45.2	25.8
Rural Health Centers	42	27.9	20.7	46.1	31.6
Urban-Rural Health Centers	40	28.9	27.5	43.8	33.4
Health Posts	63	32.6	20.5	32	29.4
Health Houses	616	23.5	19.6	41.8	28.3
Total	805	23.8	20.2	42.3	28.7

In the structural safety section, the average score of structural safety was equal to 20.2 in all healthcare facilities while the non-structural safety obtained the highest average score. However, the average score was approximately equal to 42.3 in both assessments of technical and general sub-sections of the non-structural safety section (Table 2).

**Table 2:** The Level of Structural and Non-structural Safety Components of Healthcare Facilities against Disasters and Emergencies, Kurdistan, Iran

Category	Scale	Score (%)
Structural safety	Coordination for structural safety assessment	21
	Change of structural resistance after the accident	19.6
	Assessment of structural vulnerability after the accident	21.3
	Structural Vulnerability	19.6
	Measures after structural evaluation	19.6
	Total Structural safety	20.2
Non-structural safety	General Section	41.6
	Technical Section	43
	Total Non-Structural safety	42.3

## Discussion

Our study indicated that the greatest hazards threatening the PHC facilities were climatic, biological, and geological hazard. However, according to the studies conducted in Iran, the most common natural hazards of the country have been geological and climatic [27, 28]. Specifically, the results of our study are in line with the increase in climatic [29] and biological [30] hazards in the world due to climate change. The growth in hazard groups profoundly influences people’s health and health systems [30]. A 10-year retrospective study about safety assessment of 1401 PHC centers in Iran, 2013 indicated that more than 140 PHC facilities were annually influenced by natural disasters [27]. Additionally, Radovic et al. stated that the safety of health facilities was suffered from climatic hazards in South East Europe including Croatia and Serbia in 2012 [31].

The total safety of PHC facilities in this study was equal to 28.7%. Among PHC facilities that was assessed 4.9% were in high safety, 53.9% were of moderate safety, and 41.2% were categorized in low safety. Therefore, only a very small percentage of PHC facilities were highly safe. In this study, the average safety score of the assessed PHC facilities was approximately 30 out of 100. Although this finding is in line with the average safety score of 16,078 PHC centers that measured in Iran, 2015 [32], but it is lower than the obtained safety score of PHC centers in the study in Ahwaz in 2017 [33]. A few international studies have assessed the safety of health facilities using the same tool used in this study. For instance, the general safety of 41 hospitals with the applied tool was assessed 81% in china [34] or in another study; the preparedness of rural healthcare facilities in the United States, was estimated as 78% [35]. The measured rate of safety in these studies is higher than that in this study (28.7%).

The functional preparedness of PHC facilities in this study was not acceptable and was low in comparison to the functional preparedness of PHC facilities in Ahwaz study in 2017 [33]. Moreover, although the item of financial affairs in functional preparedness of PHC facilities had the lowest score in this study (16.5), it was higher than the assessed rate across Iran (11.9) [32].

These findings were obtained in the normal situation but disasters heavily influence the performance of PHC facilities and their continuity of health services delivery to affected population [4]. Since 2001, the United States of America has made a considerable investment on promoting the preparedness of public health systems when disasters or emergencies occur [36]. This investment plays an essential role in improving the resilience of PHC facilities when disasters occur [37].

One of the elements of preparedness programs against disasters is to supply equipment [38]. Right equipment is needed to deliver the right care in the right time in the right place. The results of this study indicated that preparedness of provincial PHC facilities was weak. While, in a study conducted on evaluating the preparedness of Jordan hospitals, all the evaluated hospitals were well prepared in terms of equipment [4].

The score indicating preparedness of personnel by training and exercises in this study was very low (22.1). This result is similar with the study that was conducted on evaluating the safety of nine health and treatment centers in Indonesia in 2011in which only the personnel of two centers had been trained to be prepared for disasters and the heads of these centers were totally unaware of this training [39]. Another study on evaluating the preparedness of hospitals in Jordan in 2017 showed that one of the problems was the discontinuity in implementing training programs [4]. However, one of the strategies of improving response in PHC facilities is continuous training of personnel and volunteers. Accordingly, in Eastern Europe, implementation of the training programs of rescue and emergency evacuation when disasters occur has become obligatory in their health systems [31]. The preparedness of rapid response team in PHC facilities was equal to 48.1, which was higher than the preparedness at the national level (23.6) [32]. Having professional, experienced, active, and up-to-date teams at the scene of disaster is one of the key aspects of checking the quality-of-service delivery and protecting PHC facilities when disasters occur [40]. Therefore, training rapid response teams is necessary and having such teams is one of the components of measuring functional preparedness of these PHC facilities [41]. The preparedness of PHC facilities in the environmental health area in this study gained an acceptable score (33.7). According to the key role of environmental health in health facility preparedness, the more score in this area will result in preparedness improvement and effective health facility response when disasters occur [42].

The average score of structural safety was not acceptable. it was even lower than the structural safety rate of hospitals in Iran [21]. This result confirms the findings of the study conducted by Ardalan et al. about the vulnerability of health facilities with focus on rural health centers at time of disasters [9]. In the safety evaluation of health facilities in Eastern Europe in 2010, one of the major challenges of assessed healthcare facilities was the structural safety which was mainly related to the oldness of buildings and lack of proper renovation measures [31]. In a study of 41 Chinese hospitals, the level of structural safety was high, while the result of structural safety was classified in low category in the present study [34]. Specifically, structural safety represents the structure’s resistance to external forces [40] and it is one of the essential elements in the increase of health facilities preparedness when disasters occur [41].

The strongest point of this assessment was in the area of non-structural safety that obtained a higher score in comparison to structural safety and functional preparedness. The non-structural safety of PHC facilities under assessment was classified in the moderate safety category in this study. The reason might be attributed to the fact that the non-structural safety can be improved by taking measures with low cost such as moving or removing the objects from unsafe places. Although the non-structural safety score of PHC facilities in Kurdistan Province was lower than the assessed hospitals in this dimension in Tehran [15], the aim of improving non-structural safety in PHC facilities is to guarantee the safety of people and equipment. Improving nonstructural safety affects continuing service delivery and emergency rehabilitation measures in disasters and emergencies [41]. Inappropriate level of non-structural safety can impose heavy cost to the health system and even result in paralysis of the service provision, when it is strongly required [40, 41].

The limitation of this study was about structural and functional estimation. Calculating quantitative, and measurable structural safety score requires specific geological examination of the buildings ground foundation. Such investigation was neither available nor affordable for research team. Furthermore, the functionality of health system was measured by asking related questions and inspecting relevant documents and evidence, which might be influenced by personal perceptions. The precise estimation of functionality could be measured during a disaster or by doing simulation exercises [43, 44].

## Conclusion

The Safety of healthcare facilities has an undeniable effect on the level of preparedness and resilience against disasters and emergencies. Unacceptable level of healthcare facilities preparedness influences the continuity of service delivery to affected people from disasters. In addition, the majority of population depend on these PHC facilities for receiving governmental health services.

Considering the obtained safety score derived from different safety aspects in evaluated PHC facilities in Kurdistan province of Iran, the national and provincial decision-makers and policymakers should make right decisions for improving the preparedness of healthcare facilities. Adopting appropriate policies for improving the structural safety such as sufficient budgeting, investing in constructing new healthcare buildings and retrofitting the existing facilities are recommended. Additionally, strengthening the intersectional and intra-sectional coordination, training the personnel and people in charge of the management programs of disaster risk mitigation, and organizing the periodic exercises are suggested for increasing the functional preparedness of healthcare facilities.

## Supplementary Information

**Additional file 1.** Disaster Safety Assessment, Healthcare Facilities Checklist. this checklist used to evaluate four sections of functional, structural, non-structural, and total safety of healthcare facilities.

**Additional file 2.**

## Data Availability

The datasets used in the current study are available from the corresponding author on reasonable request.

## Abbreviations

WHO: World Health Organization
PHC: Primary Health Care
