# Supplementary material for: Disaster safety assessment of primary healthcare facilities: a cross-sectional study in Kurdistan province of Iran
Source: BMC Emerg Med. 2021 Feb 23;21:23. doi: 10.1186/s12873-021-00417-3 (PMC7903750; doi:10.1186/s12873-021-00417-3)
Supplement: Supplementary file 1 — Additional file 1. Disaster Safety Assessment, Healthcare Facilities Checklist. this checklist used to evaluate four sections of functional, structural, non-structural, and total safety of healthcare facilities. [file 12873_2021_417_MOESM1_ESM.docx]

**Disaster Safety Assessment**

**Healthcare Facilities Checklist**

**Module 1:** Hazards affecting the safety of the healthcare facilities

and the role of the healthcare facilities in emergency and disaster management

| **1.1 Hazards** | | **Hazard Level** | | | | | | | **Should the healthcare facilities be prepared to respond to this hazard?**  **If yes, mark the box.** | | **Observations (evaluator’s comments)** | |
| --- | --- | --- | --- | --- | --- | --- | --- | --- | --- | --- | --- | --- |
|  |  | **No hazard** | | **Hazard level** | | | | |  |  |  |  |
|  |  |  |  | LOW | | AVERAGE | HIGH | |  |  |  |  |
| **1.1.1 Geological hazards** | | | | | | | | | | | | |
| **1. Earthquakes** | |  | |  | |  |  | |  | |  | |
| **2. Volcanic activity and eruption** | |  | |  | |  |  | |  | |  | |
| **3. Dry mass movement − landslides** | |  | |  | |  |  | |  | |  | |
| **4. Tsunamis** | |  | |  | |  |  | |  | |  | |
| **5. Other geological hazards (e.g. rockfalls, subsidence, debris and mudflows** | |  | |  | |  |  | |  | |  | |
| **1.1.2 Hydro-meteorological hazards** | | | | | | | | | | | | |
| **1.1.2.1 Meteorological hazards** | | | | | | | | | | | | |
| **6. Cyclones/hurricanes/typhoons** | |  |  |  | | |  | |  | |  | |
| **7. Tornadoes** | |  |  |  | | |  | |  | |  | |
| **8. Local storms** | |  |  |  | | |  | |  | |  | |
| **9. Other meteorological hazards (e.g. sand-storms, wind gusts)** | |  |  |  | | |  | |  | |  | |
| **1.1.2.2 Hydrological hazards** | | | | | | | | | | | | |
| **10. River floods** | |  |  |  | | |  | |  | |  | |
| **11. Flash floods** | |  |  |  | | |  | |  | |  | |
| **12. Storm surge** | |  |  |  | | |  | |  | |  | |

| **13. Wet mass movements – landslides** | | |  |  |  |  |  | | |  |
| --- | --- | --- | --- | --- | --- | --- | --- | --- | --- | --- |
| **14. Other hydrological hazards (e.g. high tides, avalanches, coastal floods)** | | |  |  |  |  |  | | |  |
| **1.1.2.3 Climatological hazards** | | | | | | | | | | |
| **15. Extreme temperature (e.g. heat wave, cold wave, extreme winter conditions – dzud)** | | |  |  |  |  |  | | |  |
| **16. Wildfires (e.g. forests, croplands, populated areas)** | | |  |  |  |  |  | | |  |
| **17. Drought** | | |  |  |  |  |  | | |  |
| **18. Other climatological hazards including those attributable to climate change (e.g. sea-level rise)** | | |  |  |  |  |  | | |  |
| **1.1.3 Biological hazards** | | | | | | | | | | |
| **19. Epidemics, pandemics and emerging diseases** | | |  |  |  |  | |  | |  |
| **20. Foodborne outbreaks** | | |  |  |  |  | |  | |  |
| **21. Pest attacks (e.g. infestations)** | | |  |  |  |  | |  | |  |
| **22. Other biological hazards** | | |  |  |  |  | |  | |  |
| **Human-made hazards** | | | | | | | | | | |
| **1.1.4 Technological hazards** | | | | | | | | | | |
| **23. Industrial hazards (e.g. chemical, radiological)** | | |  |  |  |  | |  | |  |
| **24. Fires (e.g. building)** | | |  |  |  |  | |  | |  |
| **25. Hazardous materials (chemi- cal, biological, radiological)**  Refer to local hazard maps or other hazard informa- tion on hazardous materials (incidents and spills) inside and outside the healthcare facilities and any past incidents involving hazardous material spills or leaks, and rate the hazard- ous material hazard for the healthcare facilities and the potential  contamination of its systems. Determine whether the healthcare facilities should be prepared to respond to an emergency or disaster due to hazardous materials (based on exposure of the catchment population or the specialized role of the healthcare facilities for the treatment of patients exposed to hazard- ous materials). | **Chemical** | |  |  |  |  | |  |  | |
|  | **Biological** | |  |  |  |  | |  |  | |
|  | **Radiological** | |  |  |  |  | |  |  | |
| **26. Power outages** | | |  |  |  |  | |  |  | |
| **27. Water supply disruption** | | |  |  |  |  | |  |  | |
| **28. Transportation incidents (e.g. air, road, rail, water transport)** | | |  |  |  |  | |  |  | |
| **29. Other technological hazards (e.g. air pollution, structural collapses, food/water contamination, nuclear)** | | |  |  |  |  | |  |  | |
| **1.1.5 Societal hazards** | | | | | | | | | | |
| **30. Security threat to healthcare facilities building and staff** | | |  |  |  |  | |  |  | |
| **31. Armed conflicts** | |  | |  |  |  | |  |  | |
| **32. Civil unrest (including demonstrations)** | |  | |  |  |  | |  |  | |
| **33. Mass gathering events** | |  | |  |  |  | |  |  | |
| **34. Displaced populations** | |  | |  |  |  | |  |  | |
| **35. Other societal hazards (e.g. explosions, terrorism)** | |  | |  |  |  | |  |  | |
| **1.2 Geotechnical properties of soils** | | | | | | | | | | |
| **36. Liquefaction** | |  | |  |  |  | |  |  | |
| **37. Clay soils** | |  | |  |  |  | |  |  | |
| **38. Unstable slopes** | |  | |  |  |  | |  |  | |

Comments on the results of Form 2, Module 1: ........................................................................................................................................................................................................................................

....................................................................................................................................................................................................................................................................................................................................................

...................................................................................................................................................................................................................................................................................................................................................

Name/signature of evaluator(s).............................................................................................................................................................................................................................................................................

**Module 2:** Functional Preparedness

| **2.1 Coordination of emergency and disaster man- agement activities** | **Safety level** | | | **Observations (evaluators’ comments)** |
| --- | --- | --- | --- | --- |
|  | Low | Average | High |  |
| **39. Healthcare facilities Emergency/Disaster Committee**  Safety ratings: Low = Committee does not exist, or 1–3 departments or disciplines represented; Average = Committee exists with 4–5 depart- ments or disciplines represented, but is not fulfilling functions effectively; High = Committee exists with 6 or more departments or disciplines represented and is fulfilling functions effectively. |  |  |  |  |
| **40. Committee member responsibilities and training**  Safety ratings: Low = Committee does not exist or members are untrained and responsibilities not assigned; Average = Members have received training and have been officially assigned; High = All members are trained and are actively fulfilling their roles and responsibilities. |  |  |  |  |
| **41. Designated emergency and disaster management coordinator** Safety ratings: Low = There is no staff member who has been assigned responsibilities as the emergency/disaster management coordinator; Average = Emergency/disaster management coordination tasks have been assigned to a staff member, but it is not his/her main task; High = A staff member is assigned the emergency and disaster management coordination responsibilities as his/her main task, is fulfilling the role of implementing the healthcare facilities’s preparedness programme. |  |  |  |  |
| **42. Preparedness programme for strengthening emergency and disas- ter response and recovery**  Safety ratings: Low = A programme for strengthening preparedness, re- sponse and recovery does not exist or, if it exists, no preparedness activi- ties are being implemented; Average = A programme for strengthening preparedness, response and recovery exists and some activities are being implemented; High = A programme for strengthening preparedness, response and recovery is being fully implemented under the leadership of the Healthcare facilities Emergency/Disaster Committee. |  |  |  |  |
| **43. Healthcare facilities incident management system**  Safety ratings: Low = No arrangements for healthcare facilities incident management exist; Average = Staff assigned to key healthcare facilities incident management positions but with no written procedures to operationalize its functions; High = Healthcare facilities incident management procedures exist and are fully operational with properly trained personnel to assume different coordina- tion roles and responsibilities. |  |  |  |  |
| **44. Emergency Operations Centre (EOC)**  Safety ratings: Low = The EOC is not designated or is in an unsafe or insecure location; Average = The designated EOC is in a safe, secure and accessible location, but would have limited operational capacity immedi- ately in an emergency; High = The EOC is in a safe, secure, and accessible location with immediate operational capacity. |  |  |  |  |
| **45. Coordination mechanisms and cooperative arrangements with local emergency/disaster management agencies**  Safety ratings: Low = No arrangements exist; Average = Arrangements exist but are not fully operational; High = Arrangements exist and are fully operational. |  |  |  |  |
| **46. Coordination mechanisms and cooperative arrangements with the health-care network**  Safety ratings: Low = No arrangements exist; Average = Arrangements exist but are not fully operational; High = Arrangements exist and are fully operational. |  |  |  |  |

| **2.2 Healthcare facilities emergency and disaster response and recovery planning** | **Safety level** | | | **Observations (evaluators’ comments)** |
| --- | --- | --- | --- | --- |
|  | Low | Average | High |  |
| **47. Healthcare facilities emergency or disaster response plan**  Safety ratings: Low = Plan is not documented; Average = Documented plan is complete, but is not easily accessible, not up to date (more than 12 months since the last update); High = Plan is complete, easily accessible, reviewed/updated at least annually, and resources are available to imple- ment the plan. |  |  |  |  |
| **48. Healthcare facilities hazard-specific subplans**  Safety ratings: Low = Hazard-specific response subplans are not docu- mented; Average = Documented plans are complete but not easily ac- cessible, not up to date (more than 12 months since last review/update); High = Documented plans are complete, reviewed/updated at least annually, and resources are available to implement the plans. |  |  |  |  |
| **49. Procedures to activate and deactivate plans**  Safety ratings: Low = Procedures do not exist or exist only as a document; Average = Procedures exist, personnel have been trained, but procedures are not updated or tested annually; High = Up-to-date procedures exist, personnel have been trained, and procedures have been tested at least annually. |  |  |  |  |
| **50. Healthcare facilities emergency and disaster response plan exercises, evalua- tion and corrective actions**  Safety ratings: Low = Response plan and subplans have not been tested; Average = Response plan or subplans are tested, but are not tested at least annually; High = Response plan or subplans are tested at least annu- ally and updated according to the exercise results. |  |  |  |  |
| **51. Healthcare facilities recovery plan**  Safety ratings: Low = Recovery plan is not documented; Average = Docu- mented plan is complete, but not easily accessible, not up-to-date (more than 12 months since last review/update); High = Documented plan is complete, easily accessible, and reviewed/updated at least annually. |  |  |  |  |
| **2.3 Communication and information management** | **Safety level** | | | **Observations (evaluators’ comments)** |
|  | Low | Average | High |  |
| **52. Emergency internal and external communication**  Safety ratings: Low = Central internal and external communication system functions inconsistently or incompletely; operators are not trained in emergency communication; Average = System functions appropriately, operators have received some training in emergency communication, tests are not conducted at least annually; High = System functions com- pletely and operators are fully trained in emergency use, and tests of the system are conducted at least annually. |  |  |  |  |
| **53. External stakeholder directory**  Safety ratings: Low = Directory of external stakeholders does not exist; Average = Directory exists but is not current (more than 3 months since it was updated); High = Directory is available, is up to date and is held by key emergency response staff. |  |  |  |  |
| **54. Procedures for communicating with the public and media** Safety ratings: Low = Procedures do not exist, no spokesperson nomi- nated; Average = Procedures exist and nominated spokespersons have been trained; High = Procedures exist, nominated spokespersons have been trained, and procedures have been tested at least annually. |  |  |  |  |
| **55. Management of patient information**  Safety ratings: Low = Procedures for emergency situations do not exist; Average = Procedures for emergency situations exist and personnel have been trained but no resources are available; High = Procedures for emer- gency situations exist, personnel have been trained, and resources are in place for implementation. |  |  |  |  |

| **2.4 Human resources** | **Safety level** | | | **Observations (evaluators’ comments)** |
| --- | --- | --- | --- | --- |
|  | Low | Average | High |  |
| **56. Staff contact list**  Safety ratings: Low = Contact list does not exist; Average = List exists, but is not current (more than 3 months since it was updated); High = List is available and up to date. |  |  |  |  |
| **57. Staff availability**  Safety ratings: Low = Less than 50% of staff are available to run each department adequately; Average = 50−80% of staff are available; High = 80−100% of staff are available. |  |  |  |  |
| **58. Mobilization and recruitment of personnel during an emergency or disaster**  Safety ratings: Low = Procedures do not exist or exist only in a document; Average = Procedures exist and personnel have been trained, but the human resources for an emergency situation are not available; High = Procedures exist, personnel have been trained, and the human resources are available to meet anticipated needs in an emergency. |  |  |  |  |
| **59. Duties assigned to personnel for emergency or disaster response and recovery**  Safety ratings: Low = Emergency assignments do not exist or are not documented; Average = Duties are identified, some (but not all) person- nel receive written assignments or training; High = Written duties are assigned, and training or an exercise is conducted for all personnel at least annually. |  |  |  |  |
| **60. Well-being of healthcare facilities personnel during an emergency or disaster** Safety ratings: Low = A designated space and measures do not exist; Average = Space has been designated, but measures cover less than 72 hours; High = Measures are ensured for at least 72 hours. |  |  |  |  |
| **2.5 Logistics and finance** | **Safety level** | | | **Observations (evaluators’ comments)** |
|  | Low | Average | High |  |
| **61. Agreements with local suppliers and vendors for emergencies and disasters**  Safety ratings: Low = No arrangements exist; Average = Arrangements exist, but are not fully operational; High = Arrangements exist and are fully operational. |  |  |  |  |
| **62. Transportation during an emergency**  Safety ratings: Low = Ambulances and other vehicles and modes of transportation are not available; Average = Some vehicles are available, but not in sufficient numbers for a major emergency or disaster; High = Appropriate vehicles in sufficient numbers are available during emergen- cies/disasters. |  |  |  |  |
| **63. Food and drinking-water during an emergency**  Safety ratings: Low = Procedures for food and drinking-water for emer- gencies are non-existent; Average = Procedures exist, food and drinking- water is guaranteed for less than 72 hours; High = Food and drinking- water for emergencies is guaranteed for at least 72 hours. |  |  |  |  |
| **64. Financial resources for emergencies and disasters**  Safety ratings: Low = Emergency budget or mechanism to access emergency funds is not in place; Average = Funds are budgeted and mechanisms are available but cover less than 72 hours; High = Sufficient funds are guaranteed for 72 hours or more. |  |  |  |  |

| **2.6 Patient care and support services** | **Safety level** | | | **Observations (evaluators’ comments)** |
| --- | --- | --- | --- | --- |
|  | Low | Average | High |  |
| **65. Continuity of emergency and critical care services**  Safety ratings: Low = Procedures do not exist or exist only as a document; Average = Procedures exist, personnel have been trained but would not be available at all times; High = Procedures exist, personnel have been trained, and resources are available to implement procedures at maxi- mum healthcare facilities capacity for emergency and disaster situations at all times. |  |  |  |  |
| **66. Continuity of essential clinical support services**  Safety ratings: Low = Procedures do not exist or exist only as a document; Average = Procedures exist and personnel have been trained but would not be available at all times; High = Procedures exists, personnel have been trained, and resources are available to implement procedures at maximum healthcare facilities capacity for emergency and disaster situations at all times. |  |  |  |  |
| **67. Expansion of usable space for mass casualty incidents**  Safety ratings: Low = Space for expansion has not been identified; Aver- age = Space has been identified; equipment, supplies and procedures are available to carry out the expansion and staff have been trained, but testing has not been conducted; High = Procedures exist and have been tested, personnel have been trained, and equipment, supplies and other resources are available to carry out the expansion of space. |  |  |  |  |
| **68. Triage for major emergencies and disasters**  Safety ratings: Low = Designated triage location or procedures do not exist; Average = Triage location and procedures exist and personnel have been trained, but procedures have not been tested for emergency and disaster situations; High = Location and procedures exist and have been tested, personnel have been trained, and resources are in place to imple- ment at maximum healthcare facilities capacity in emergency and disaster situations. |  |  |  |  |
| **69. Triage tags and other logistical supplies for mass casualty incidents** Safety ratings: Low = Nonexistent; Average = Supply covers less than 72 hours of maximum healthcare facilities capacity; High = Supply guaranteed for at least 72 hours of maximum healthcare facilities capacity. |  |  |  |  |
| **70. System for referral, transfer and reception of patients**  Safety ratings: Low = Procedures do not exist or exist only as a document; Average = Procedures exist and personnel have been trained, but pro- cedures have not been tested for emergency or disaster situations; High  = Procedures exist and have been tested, personnel have been trained, and resources are available to implement measures at maximum healthcare facilities capacity in emergency or disaster situations. |  |  |  |  |
| **71. Infection surveillance, prevention and control procedures**  Safety ratings: Low = Policies and procedures do not exist; standard pre- cautions for infection prevention and control are not followed routinely; Average = Policies and procedures exist, standard precautions are rou- tinely followed, personnel have been trained, but the level of resources required for emergency and disaster situations, including epidemics, is not available; High = Policies and procedures exist, infection prevention and control measures are in place, personnel have been trained, and resources are available to implement measures at maximum healthcare facilities capacity in emergency and disaster situations. |  |  |  |  |
| **72. Psychosocial services**  Safety ratings: Low = Procedures do not exist or exist only as a docu- ment; Average = Procedures exist and personnel have been trained, but the level of resources required for emergency and disaster situations is not available; High = Procedures exist, personnel have been trained, and resources are available for implementation of procedures at maximum healthcare facilities capacity in emergency and disaster situations. |  |  |  |  |

| **73. Post-mortem procedures in a mass fatality incident**  Safety ratings: Low = Procedures for a mass fatality incident do not exist or exist only as a document; Average = Procedures exist and personnel have been trained, but the level of resources required for emergency and disaster situations is not available; High = Procedures exist, personnel have been trained, and resources are available for implementation of procedures at maximum healthcare facilities capacity in emergency and disaster situations. |  |  |  |  |
| --- | --- | --- | --- | --- |
| **2.7 Evacuation, decontamination and security** | **Safety level** | | | **Observations (evaluators’ comments)** |
|  | Low | Average | High |  |
| **74. Evacuation plan**  Safety ratings: Low = Plan does not exist or exists only as a document; Average = Plan exists and personnel have been trained in procedures, but tests are not conducted regularly; High = Plan exists, personnel have been trained, and evacuation drills are held at least annually. |  |  |  |  |
| **75. safe**ty ratings: Low = No personal protective equipment is available for immediate use by healthcare facilities staff, or no decontamination area exists;  Average = Personal protective equipment is available for immediate use, decontamination areas are established, staff training and drills are not conducted annually; High = Personal protective equipment is available for immediate use, decontamination areas are established and personnel are trained and tested at least annually. |  |  |  |  |
| **76. Personal protection equipment and isolation for infectious dis- eases and epidemics**  Safety ratings: Low = No personal protective equipment is available for immediate use by healthcare facilities staff, or no isolation area exists; Average = Sup- ply is available for immediate use, but is sufficient for less than 72 hours of maximum healthcare facilities capacity, isolation areas are established, staff training and testing of procedures are not conducted annually; High = Supply is guaranteed for at least 72 hours of maximum healthcare facilities capacity and alter- nate sources are in place for resupply, isolation areas are established, staff training and testing of procedures are conducted at least annually. |  |  |  |  |
| **77. Emergency security procedures**  Safety ratings: Low = Emergency security procedures do not exist or exist only as a document; Average = Documented procedures exist and per- sonnel have been trained in emergency security procedures but testing is not conducted at least annually; High = Personnel are trained and tests of the documented procedures are held at least annually. |  |  |  |  |
| **78. Computer system network security**  Safety ratings: Low = The healthcare facilities does not have a computer security sys- tem plan and procedures in place; Average = The healthcare facilities has a basic cyber security plan in place but it is not monitored and updated regularly; High = The healthcare facilities has a cyber security plan in place and it is updated regularly. |  |  |  |  |

Comments on the results of Form 2, Module 3.

................................................................................................................................................................................................................................................................................................................

................................................................................................................................................................................................................................................................................................................

................................................................................................................................................................................................................................................................................................................

................................................................................................................................................................................................................................................................................................................

................................................................................................................................................................................................................................................................................................................

Name/signature of evaluator(s).........................................................................................................................................................................................................................................

**Module 3:** Nonstructural safety

| **3.1. Architectural safety** | **Safety level** | | | **Observations (evaluators’ comments)** |
| --- | --- | --- | --- | --- |
|  | Low | Average | High |  |
| **79. Major damage and repair of nonstructural elements**  *Safety ratings: Low = Major damage and no repairs completed; Average = Moderate damage, building only partially repaired; High = Minor or no dam- age, or building fully repaired.*  *IF SUCH AN EVENT HAS NOT OCCURRED IN THE VICINITY OF THE HEALTHCARE FACILITIES, LEAVE BOXES BLANK AND PROVIDE COMMENT.* |  |  |  |  |
| **80. Condition and safety of doors, exits and entrances**  *Safety ratings: Low = Doors, exits and entrances in poor condition, subject to damage which would impede the function of this and other elements, systems or operations; entrance width is less than 115cm; Average = In fair*  *condition, subject to damage but damage would not impede the function of this and other elements, systems or operations; or entrance width is less than 115cm; High = In good condition, no or minor potential for damage that would impede the function of this and other elements, systems or operations; and entrance width is equal to or larger than 115cm.* |  |  |  |  |
| **81. Condition and safety of windows and shutters**  *Safety ratings: Low = Windows and shutters in poor condition, subject to damage which would impede the function of this and other elements, systems or operations (e.g. weak protective glazing); Average = In fair condi- tion, subject to damage but damage would not impede the function of this and other elements, systems or operations; High = In good condition, no or minor potential for damage that would impede the function of this and other elements, systems or operations; protective glass (e.g. polycarbonate glazing, blast film) has been added in critical wards.* |  |  |  |  |
| **82. Condition and safety of other elements of the building envelope (e.g. outside walls, facings)**  *Safety ratings: Low = Building envelope in poor condition, subject to damage which would impede the function of this and other elements, systems or operations; Average = In fair condition, subject to damage but damage would not impede the function of this and other elements, systems or operations; High = In good condition, no or minor potential for damage that would impede the function of this and other elements, systems or operations.* |  |  |  |  |
| **83. Condition and safety of roofing**  *Safety ratings: Low = Roofing in poor condition, subject to damage which would impede the function of this and other elements, systems or operations; Average = In fair condition, subject to damage but damage to element(s) would not impede the function of this and other elements, systems or opera- tions; High = In good condition, no or minor potential for damage that would impede the function of this and other elements, systems or operations.* |  |  |  |  |
| **84. Condition and safety of railings and parapets**  *Safety ratings: Low = Railings and parapets in poor condition, subject to dam- age which would impede the function of this and other elements, systems or operations; Average = Subject to damage but damage to element(s) would not impede the function of this and other elements, systems or operations; High = No or minor potential for damage that would impede the function of this and other elements, systems or operations.* |  |  |  |  |
| **85. Condition and safety of perimeter walls and fencing**  *Safety ratings: Low = Perimeter walls and fencing in poor condition, subject to damage which would impede the function of this and other elements, systems or operations; Average = In fair condition, subject to damage but damage to element(s) would not impede the function of this and other elements, systems or operations; High = In good condition, no or minor potential for damage that would impede the function of this and other elements, systems or operations.* |  |  |  |  |

Continue >>

| **86. Condition and safety of other architectural elements (e.g. cornices, ornaments, chimneys, signs)**  *Safety ratings: Low = Other architectural element(s) in poor condition, subject to damage which would impede the function of this and other elements, systems or operations; Average = In fair condition, element(s) are subject to damage but damage would not impede the function of this and other elements, systems or operations; High = In good condition, no or minor potential for damage that would impede the function of this and other elements, systems or operations.* |  |  | |  |  |
| --- | --- | --- | --- | --- | --- |
| **87. Safe conditions for movement outside the healthcare facilities buildings**  *Safety ratings: Low = Obstacles or damage to structure or road and walkways will impede vehicle and pedestrian access to buildings or endanger pedes- trians; Average = Obstacles or damage to structure or road and walkways*  *will not impede pedestrian access, but will impede vehicle access; High = No obstacles, or potential for only minor or no damage that will not impede pedestrian or vehicle access.* |  |  | |  |  |
| **88. Safe conditions for movement inside the building (e.g. corridors, stairs)** *Safety ratings: Low = Obstacles and damage to element(s) will impede move- ment inside the building and endanger occupants; Average = Obstacles or damage to elements will not impede movement of people but will impede movement of stretchers, wheeled equipment; High = No obstacles, potential for no or minor damage which will not impede movement of people or wheeled equipment.* |  |  | |  |  |
| **89. Condition and safety of internal walls and partitions**  *Safety ratings: Low = Internal walls and partitions in poor condition, subject to damage which would impede the function of this and other elements, systems or operations; Average = In fair condition, element(s) are subject to damage but damage would not impede the function of this and other ele- ments, systems or operations; High = In good condition, no or minor potential for damage that would impede the function of this and other elements, systems or operations.* |  |  | |  |  |
| **90. Condition and safety of false or suspended ceilings**  *Safety ratings: Low = False or suspended ceilings in poor condition, subject to damage which would impede the function of this and other elements, systems or operations; Average = In fair condition, element(s) subject to damage but damage would not impede the function of this and other elements, systems or operations; High = In good condition, no or minor potential for damage that would impede the function of this and other elements, systems or operations.*  *IF THE HEALTHCARE FACILITIES DOES NOT HAVE FALSE OR SUSPENDED CEILINGS, LEAVE BOXES BLANK.* |  |  | |  |  |
| **91. Condition and safety of the elevator system**  *Safety ratings: Low = Elevator system in poor condition, subject to damage which would impede the function of this and other elements, systems or operations; Average = In fair condition, element(s) subject to damage but damage would not impede the function of this and other elements, systems or operations; High = In good condition, no or minor potential for damage that would impede the function of this and other elements, systems or operations.*  *IF THERE ARE NO ELEVATORS, LEAVE BOXES BLANK AND PROVIDE COMMENT.* |  |  | |  |  |
| **92. Condition and safety of stairways and ramps**  *Safety ratings: Low = In poor condition, subject to damage or there are ob- stacles, which would impede the function of this and other elements, systems or operations; Average = In fair condition, subject to damage but damage and obstacles would not impede the function of this and other elements, systems or operations; High = In good condition, no obstacles, potential for no or minor damage that would impede the function of this and other elements, systems or operations.*  *IF THERE ARE NO STAIRS AND RAMPS, LEAVE BOXES BLANK AND PROVIDE COMMENT.* |  |  | |  |  |
| **93. Condition and safety of floor coverings**  *Safety ratings: Low = Floor coverings in poor condition, subject to damage which would impede the function of this and other elements, systems or opera- tions; Average = In fair condition, subject to damage but damage would not im- pede function; High = In good condition, no or minor potential for damage that would impede the function of this and other elements, systems or operations.* |  |  | |  |  |
| **3.2 Infrastructure protection, access and physical security** | **Safety level** | | | | **Observations (evaluators’ comments)** |
|  | Low | Average | | High |  |
| **94 Location of healthcare facilities’s critical services and equipment in the healthcare facilities in relation to local hazards**  *Safety ratings: Low = No protection measures taken; subject to damage, failure and disruption of critical services and healthcare facilities operations in emergen- cies and disasters; Average = Partial measures to protect critical services from local hazards are taken; subject to damage with some disruption of critical services and healthcare facilities operations in emergencies or disasters; High = Many measures are taken to protect critical services; high probability that critical services and healthcare facilities will operate with no or limited disruption in emergencies and disasters.* |  |  | |  |  |
| **95. Healthcare facilities access routes**  *Safety ratings: Low = Access routes subject to obstacles and damage that would impede access and the function of other elements, systems or opera- tions; Average = Access routes subject to some obstacles and damage that would not impede access and function; High = No or minor potential for obstacles or damage that would impede access and the function of other elements, systems or operations.* |  |  | |  |  |
| **96. Emergency exits and evacuation routes**  *Safety ratings: Low = Exit and evacuation routes are not clearly marked and many are blocked; Average = Some exit and evacuation routes are marked and most are clear of obstacles; High = All exit and evacuation routes are clearly marked and free of obstacles.* |  |  | |  |  |
| **97. Physical security of building, equipment, staff and patients**  *Safety ratings: Low = No measures are in place; Average = Some physical security protection is in place (e.g. locked storage for supplies and equipment, asset tracking and inventory control); High = Wide range of security measures in place (e.g. design and layout, physical barriers, access control and door security systems, locked storage for supplies and equipment).* |  |  | |  |  |
| **3.3 Critical systems** | **Safety level** | | | | **Observations (evaluators’ comments)** |
|  | Low | Average | | High |  |
| **3.3.1 Electrical systems** | | | | | |
| **98. Capacity of alternate sources of electricity (e.g. generators)**  *Safety ratings: Low = Alternate source(s) is(are) missing or covers less than 30% of demand in critical areas, or can only be started manually; Average = Alternate source(s) covers 31–70% of demand in critical areas and starts au- tomatically in less than 10 seconds in critical areas; High = Alternate source(s) start(s) automatically in less than 10 seconds and cover(s) more than 70% of demand in critical areas.* |  |  | |  |  |
| **99. Regular tests of alternate sources of electricity in critical areas**  *Safety ratings: Low = Tested at full load every 3 months or more; Average*  *= Tested at full load every 1 to 3 months; High = Tested at full load at least monthly.* |  |  | |  |  |
| **100. Condition and safety of alternate source(s) of electricity**  *Safety ratings: Low = No alternate sources; generators are in poor condition, there are no protective measures; Average = Generators are in fair condition, some measures provide partial protection and security; High = Generators are in good condition, well-secured and in good working order for emergencies.* |  |  | |  |  |
| **101. Condition and safety of electrical equipment, cables and cable ducts** *Safety ratings: Low = Electrical equipment, power lines, cables and ducts are in poor condition, there are no protective measures; Average = Electrical equip- ment, power lines, cables and ducts are in fair condition; some measures provide partial protection and security; High = Electrical equipment, power lines, cables and ducts are in good condition, well-secured and in good working order.* |  |  | |  |  |
| **102. Redundant system for the local electric power supply**  *Safety ratings: Low = There is only one entrance for the local power supply; Average = There are two entrances for the local power supply; High = There are more than two entrances for the local power supply.* |  |  | |  |  |
| **103. Condition and safety of control panels, overload breaker switches and cables**  Safety ratings: Low = Control panels or other elements are in poor condi- tion, there are no protective measures; Average = Control panels or other elements are in fair condition; some measures provide partial protection; High = Control panels or other elements are in good condition, well- protected and in good working order. |  |  | |  |  |
| **104. Lighting system for critical areas of the healthcare facilities**  Safety ratings: Low = Poor level of lighting, there are no protective measures; Average = Lighting is satisfactory in the critical areas; some measures provide partial protection; High = Good levels of lighting and protection measures in place. |  |  | |  |  |
| **105. Condition and safety of internal and external lighting systems** Safety ratings: Low = Internal and external lighting systems are in poor condition, there are no protective measures; Average = In fair condition; some measures provide partial protection; High = In good condition, well-protected and in good working order. |  |  | |  |  |
| **106. External electrical systems installed for healthcare facilities usage**  Safety ratings: Low = No electrical substations installed for healthcare facilities demands; Average = Substations installed; some measures provide partial protection, but would be vulnerable to damage or disruption, do not provide enough power to the healthcare facilities; High = Electrical substations installed, well-protected, and provide enough power to the healthcare facilities in an emergency or disaster. |  |  | |  |  |
| **107. Emergency maintenance and restoration of electric power supply and alternate sources**  Safety ratings: Low = Documented procedures and maintenance/in- spection records do not exist; Average = Documented procedures exist, maintenance/inspection records are up to date, personnel have been trained, but resources are not available; High = Documented procedures exist, maintenance/inspection records are up to date, personnel have been trained, and resources are in place for implementing emergency maintenance and restoration. |  |  | |  |  |
| **3.3.2 Telecommunications systems** | | | | | |
| **108. Condition and safety of antennas**  Safety ratings: Low = Antennas and bracing in poor condition, there are no protective measures; Average = Antennas and bracing are in fair condition, some measures provide partial protection; High = Antennas and bracing are in good condition, well-secured and protection measures are in place.  IF THERE ARE NO ANTENNAS, LEAVE BOXES BLANK AND PROVIDE COMMENT. |  |  | |  |  |
| **109. Condition and safety of low- and extra-low-voltage systems (internet and telephone)**  Safety ratings: Low = Low voltage systems in poor condition, there are no protective measures; Average = Low voltage systems in fair condition, some measures provide partial protection; High = Good condition, well- secured and other protection measures in place. |  |  | |  |  |
| **110. Alternate communication systems**  Safety ratings: Low = Alternate communications systems do not exist, are in poor condition, or do not function; Average = Healthcare facilities-wide alternate communications system in fair condition, but is not tested on an annual basis; High = Alternate communication system in good condition and tested at least annually. |  |  | |  |  |
| **111. Condition and safety of telecommunications equipment and cables** Safety ratings: Low = Telecommunications equipment and cables are in poor condition; there are no protective measures; Average = Equipment and cables are in fair condition; some measures provide partial protection; High = In good condition, well-secured and protected from hazards. |  |  | |  |  |
| **112. Effect of external telecommunications systems on healthcare facilities com- munications**  Safety ratings: Low = External telecommunications systems cause major interference with healthcare facilities communications; Average = External telecommunications system cause moderate interference with healthcare facilities  communications; High = External communications cause no interference with healthcare facilities communications. |  |  | |  |  |
| **113. Safety of sites for telecommunication systems**  Safety ratings: Low = Sites for telecommunications systems are in poor condition, at high risk of failure due to hazards; there are no protective measures; Average = Sites in fair condition, some measures provide par- tial protection; High = Good condition, well-secured and other protective measures in place. |  |  | |  |  |
| **114. Condition and safety of internal communications systems**  Safety ratings: Low = Internal communications systems do not exist or are in poor condition; Average = Internal communications systems are in fair condition, but there are no alternate systems; High = Internal communi- cations and back-up systems are in good working order. |  |  | |  |  |
| **115. Emergency maintenance and restoration of standard and alternate communications systems**  Safety ratings: Low = Documented procedures and maintenance/in- spection records do not exist; Average = Documented procedures exist, maintenance/inspection records are up to date, personnel have been trained, but resources are not available; High = Documented procedures exist, maintenance/inspection records are up to date, personnel have been trained, and resources are in place for implementing emergency maintenance and restoration. |  |  | |  |  |
| **3.3.3 Water supply system** | | | | | |
| **116. Water reserves for healthcare facilities services and functions**  Safety ratings: Low = Sufficient for 24 hours or less or water tank does not exist; Average = Sufficient for more than 24 hours but less than 72 hours; High = Guaranteed to cover at least 72 hours. |  |  | |  |  |
| **117. Location of water storage tanks**  Safety ratings: Low = The site is vulnerable with high risk of failure (e.g. structural, architectural and/or system vulnerabilities); Average = The site is exposed to moderate risk of failure (e.g. structural, architectural and/or system vulnerabilities); High = The site is not exposed to visually identifi- able risks (e.g. structural, architectural and/or system vulnerabilities).  IF THE HEALTHCARE FACILITIES DOES NOT HAVE A WATER STORAGE TANK, LEAVE BOXES BLANK AND PROVIDE COMMENT. |  |  | |  |  |
| **118. Safety of the water distribution system**  Safety ratings: Low = Less than 60% are in good operational condition; Average = Between 60% and 80% are in good condition; High = Above 80% are in good condition. |  |  | |  |  |
| **119. Alternate water supply to the regular water supply**  Safety ratings: Low = Provides less than 30% of daily demand in an emer- gency or disaster scenario; Average = Provides 30−80% of daily demand in an emergency or disaster scenario; High = Provides more than 80% of daily demand in an emergency or disaster scenario. |  | |  |  |  |
| **120. Supplementary pumping system**  Safety ratings: Low = There is no back-up pump and operational capac- ity does not meet minimum daily demand; Average = Supplementary pumps are in fair condition but would not meet the minimum daily de- mand for water; High = All supplementary pumps and back-up systems are operational and would meet the minimum demand for water. |  | |  |  |  |
| **121. Emergency maintenance and restoration of water supply systems** Safety ratings: Low = Documented procedures and maintenance/in- spection records do not exist; Average = Documented procedures exist, maintenance/inspection records are up to date, personnel have been trained, but resources are not available; High = Documented procedures exist, maintenance/inspection records are up to date, personnel have been trained, and resources are in place for implementing emergency maintenance and restoration. |  | |  |  |  |
| **3.3.4 Fire protection system** | | | | | |
| **122. Condition and safety of the fire protection (passive) system** Safety ratings: Low = Element(s) are subject to damage, and damage would impede the function of this and other elements, systems or operations; Average = Element(s) are subject to damage but damage would not impede function; High = No or minor potential for damage that would impede the function of this and other elements, systems or operations. |  | |  |  |  |
| **123. Fire/smoke detection systems**  Safety ratings: Low = No system has been installed; Average = System is partially installed, or infrequently maintained and tested; High = System is installed and well-maintained and tested frequently. |  | |  |  |  |
| **124. Fire suppression systems (automatic and manual)**  Safety ratings: Low = No system has been installed; inspections do not occur; Average = System is partially installed, or system is installed, but no maintenance or testing; inspections are incomplete or outdated; High = System is fully installed and regularly maintained and tested frequently; inspections are complete and up to date. |  | |  |  |  |
| **125. Water supply for fire suppression**  Safety ratings: Low = A source of permanent supply which could be used for fire suppression does not exist; Average = A source of permanent supply of water is available for fire suppression; there is limited capacity available, and no maintenance and testing has been conducted; High = A source of permanent water supply with significant capacity for fire sup- pression is available, regularly maintained and frequently tested. |  | |  |  |  |
| **126. Emergency maintenance and restoration of the fire protection system**  Safety ratings: Low = Documented procedures and maintenance/in- spection records do not exist; Average = Documented procedures exist, maintenance/inspection records are up to date, personnel have been trained, but resources are not available; High = Documented procedures exist, maintenance/inspection records are up to date, personnel have been trained, and resources are in place for implementing emergency maintenance and restoration. |  | |  |  |  |

Continue >>

| **3.3.5 Waste management systems** | | | | |
| --- | --- | --- | --- | --- |
| **127. Safety of nonhazardous wastewater systems**  Safety ratings: Low = System for nonhazardous wastewater disposal does not exist or is in poor condition; Average = System is in fair condition, but little or no evidence of compliance and maintenance; High = Wastewater disposal system is in good condition with good capacity and evidence of compliance and maintenance. |  |  |  |  |
| **128. Safety of hazardous wastewater and liquid waste**  Safety ratings: Low = System for hazardous wastewater disposal does not exist or is in poor condition; Average = System is in fair condition but little or no evidence of compliance and maintenance; High = Disposal system has good capacity and evidence of compliance and maintenance. |  |  |  |  |
| **129. Safety of nonhazardous solid waste system**  Safety ratings: Low = System for solid waste disposal does not exist or is in poor condition; Average = System is in fair condition, but little or no evidence of compliance and maintenance; High = Disposal system is in good condition with good capacity and evidence of compliance and maintenance. |  |  |  |  |
| **130. Safety of hazardous solid waste system**  Safety ratings: Low = System for hazardous waste disposal does not exist or is in poor condition; Average = System is in fair condition but little or no evidence of compliance and maintenance; High = Disposal system is in good condition with good capacity and evidence of compliance and maintenance. |  |  |  |  |
| **131. Emergency maintenance and restoration of all types of healthcare facilities waste management systems**  Safety ratings: Low = Documented procedures and maintenance/in- spection records do not exist; Average = Documented procedures exist, maintenance/inspection records are up to date, personnel have been trained, but resources are not available; High = Documented procedures exist, maintenance/inspection records are up to date, personnel have been trained, and resources are in place for implementing emergency maintenance and restoration. |  |  |  |  |
| **3.3.6 Fuel storage systems (e.g. gas, gasoline and diesel)** | | | | |
| **132. Fuel reserves**  Safety ratings: Low = Sufficient for 24 hours or less, or fuel tank does not exist; Average = Sufficient for more than 24 hours but less than 72 hours; High = Guaranteed to cover at least 72 hours. |  |  |  |  |
| **133. Condition and safety of above-ground fuel tanks and/or cylinders** Safety ratings: Low = Tanks are in poor condition; there are no anchors or tank enclosure; tanks are not safely located with respect to hazards; Average  = Tanks are in fair condition, anchors and bracing are inadequate for major hazards; tank enclosure has some safety and security measures; High = Tanks are in good condition; anchors and bracing are in good condition for major hazards; the tank enclosure has adequate safety and security.  IF THE HEALTHCARE FACILITIES DOES NOT HAVE THESE SERVICES, LEAVE BOXES BLANK AND PROVIDE COMMENT. |  |  |  |  |
| **134. Safe location of fuel storage away from healthcare facilities buildings** Safety ratings: Low = Fuel storage is not accessible and is not located in a secure site; Average = Site in fair condition and in fair location in  relation to hazards; some measures provide partial protection; High = In good condition and good location, well-secured and other protection measures in place; fuel tanks are accessible.  IF THERE IS NO FUEL TANK, LEAVE BOXES BLANK AND PROVIDE COMMENT. |  |  |  |  |

| **135. Condition and safety of the fuel distribution system (valves, hoses, connections)**  Safety ratings: Low = Less than 60% of the system is in safe operational condi- tion; Average = between 60% and 90% of the system is in good operational condition and has automatic shut-off valves; High = More than 90% of the system is in good operational condition and has automatic shut-off valves.  IF THERE IS NO FUEL DISTRIBUTION TANK, LEAVE BOXES BLANK AND PROVIDE COMMENT. |  |  |  |  |
| --- | --- | --- | --- | --- |
| **136. Emergency maintenance and restoration of fuel reserves**  Safety ratings: Low = Documented procedures and maintenance/in- spection records do not exist; Average = Documented procedures exist, maintenance/inspection records are up to date, personnel have been trained, but resources are not available; High = Documented procedures exist, maintenance/inspection records are up to date, personnel have been trained, and resources are in place for implementing emergency maintenance and restoration. |  |  |  |  |
| **3.3.7 Medical gases systems** | | | | |
| **137. Location of storage areas for medical gases**  Safety ratings: Low = No sites reserved for medical gases, or sites for medi- cal gases are at high risk of failure due to hazards; there are no protective measures, and storage is not accessible; Average = Reserved areas in fair condition and fair location; some measures provide partial protection; High = In good condition, well-secured and other protective measures in place; storage is accessible. |  |  |  |  |
| **138. Safety of storage areas for medical gas tanks and/or cylinders** Safety ratings: Low = Medical gas tanks and cylinders in storage areas are poor condition; no protection measures, not secured; personnel are not trained to operate medical gas and fire extinguishing equipment;  Average = Medical gas tanks and cylinders in storage areas are in fair con- dition, some measures provide partial protection; the quality of anchors and braces is inadequate; personnel are trained to operate equipment; High = Good condition, well-secured and protected, anchors are of good quality for major hazards; medical gas and fire extinguishing equipment operated by qualified personnel. |  |  |  |  |
| **139. Condition and safety of medical gas distribution system (e.g. valves, pipes, connections)**  Safety ratings: Low = Less than 60% of the system is in good working condition; Average = Between 60% and 80% of the system is in good working condition; High = More than 80% of the system is in good work- ing condition. |  |  |  |  |
| **140. Condition and safety of medical gas cylinders and related equip- ment in the healthcare facilities**  Safety ratings: Low = Medical gas tanks and cylinders in healthcare facilities areas are in poor condition, no protective measures; not secured; Average = Medical gas tanks and cylinders are in fair condition; the quality of anchors and braces is inadequate; some measures provide partial protection; High = Good condition, well-secured and protected; anchors are of good quality for major hazards. |  |  |  |  |
| **141. Availability of alternative sources of medical gases**  Safety ratings: Low = Alternative sources are not available; Average = Alternative sources in place but delivery of supplies takes longer than 15 days; High = Sufficient alternative sources are available at short notice (less than 15 days). |  |  |  |  |

Continue >>

| **142. Emergency maintenance and restoration of medical gas systems** Safety ratings: Low = Documented procedures and maintenance/in- spection records do not exist; Average = Documented procedures exist, maintenance/inspection records are up to date, and personnel have been trained, but resources are not available; High = Procedures exist, mainte- nance/inspection records are up to date, personnel have been trained, and resources are in place for implementing emergency maintenance and restoration. |  |  |  |  |
| --- | --- | --- | --- | --- |
| **3.3.8 Heating, ventilation, and air-conditioning (HVAC) systems** | | | | |
| **143. Adequate location of enclosures for HVAC equipment**  Safety ratings: Low = HVAC enclosures are not accessible and they are not located in a safe site; there are no protective measures; Average = HVAC enclosures are accessible, located at a safe site; some measures provide partial protection from hazards; High = HVAC enclosures are accessible, in a safe location and protected from hazards. |  |  |  |  |
| **144. Safety of enclosures for HVAC equipment**  Safety ratings: Low = HVAC equipment is not accessible; no protection measures for safe operation and maintenance; Average = HVAC is acces- sible; some measures provide partial protection; High = HVAC equipment is accessible, wide range of protection measures in place. |  |  |  |  |
| **145. Safety and operating condition of HVAC equipment (e.g. boiler, exhaust)**  Safety ratings: Low = HVAC equipment in poor condition, not maintained; Average = HVAC equipment in fair condition; some measures provide partial protection, but no regular maintenance; High = Good condition, well-secured and protected from hazards (e.g. anchors are of good qual- ity); regular maintenance and testing of controls and alarms conducted. |  |  |  |  |
| **146. Adequate supports for ducts and review of flexibility of ducts and piping that cross expansion joints**  Safety ratings: Low = Supports are lacking and connections are rigid; Average = Supports are in fair condition or connections are flexible; High  = Supports are in good condition and connections are flexible. |  |  |  |  |
| **147. Condition and safety of pipes, connections and valves**  *Safety ratings: Low = Less than 60% of pipes are in good condition; limited protective measures against hazards; Average = Between 60% and 80% are in good condition; some measures provide partial protection against hazards; High = Above 80% are in good condition and are well-secured and protected against hazards.* |  |  |  |  |
| **148. Condition and safety of air-conditioning equipment**  Safety ratings: Low = Air-conditioning units in poor condition, not secured; Average = Air-conditioning units are in fair condition; some measures provide partial protection (e.g. quality of anchors and braces is inadequate); High = Good condition, well-secured and protected from hazards (e.g. anchors are of good quality). |  |  |  |  |
| **149. Operation of air-conditioning system (including negative pressure areas)** Safety ratings: Low = Air-conditioning system has no capability for estab- lishing zones of the healthcare facilities; Average = Air-conditioning system can estab- lish zones, but has no capacity to separate air circulating between high-risk areas and other areas of the healthcare facilities; High = Air-conditioning system can isolate air from high-risk areas; negative pressure rooms are available. |  |  |  |  |

Continue >>

| **150. Emergency maintenance and restoration of HVAC systems**  Safety ratings: Low = Documented procedures and maintenance/in- spection records do not exist; Average = Documented procedures exist, maintenance/inspection records are up to date, personnel have been trained, but resources are not available; High = Documented procedures exist, maintenance/inspection records are up to date, personnel have been trained, and resources are in place for implementing emergency maintenance and restoration. |  |  |  |  |
| --- | --- | --- | --- | --- |
| **3.4 Equipment and supplies** | **Safety level** | | | **Observations (evaluators’ comments)** |
|  | Low | Average | High |  |
| **3.4.1 Office and storeroom furnishings and equipment (fixed and movable)** | | | | |
| **151. Safety of shelving and shelf contents**  Safety ratings: Low = Shelving is not safely located (or in seismic and wind-prone areas not attached to walls in more than 20% of cases); Average = Shelving is safely located (and attached to walls in seismic and wind-prone areas) and contents are secured in 20−80% of cases; High = More than 80% of shelving and the contents of shelves are safely located, attached to walls, and contents are secured. |  |  |  |  |
| **152. Safety of computers and printers**  Safety ratings: Low = No measures to protect computers from hazards are in place; Average =Computers are in safe locations, some measures offer partial protection from hazards; High = Computers are in safe locations, well-secured and good protective measures in place. |  |  |  |  |
| **3.4.2 Medical and laboratory equipment and supplies used for diagnosis and treatment** | | | | |
| **153. Safety of medical equipment in operating theatres and recovery rooms**  Safety ratings: Low = The operating theatres are in an unsafe location, equipment is lacking or in poor condition or there are no protective mea- sures; Average = The operating theatres are in a safe location, equipment is in fair condition, and some measures provide partial protection; High = Operating theatres are in a safe location, equipment is in good condition, is well-secured and measures provide protection. |  |  |  |  |
| **154. Condition and safety of radiology and imaging equipment**  *Safety ratings: Low = The radiology and imaging equipment is not in a safe location, equipment is lacking or in poor condition, or there are no protective measures; Average = The equipment is in a safe location, is in fair condition, and some measures offer partial protection; High = Equipment is in a safe location, is in good condition, well-secured and measures provide good protection.* |  |  |  |  |
| **155. Condition and safety of laboratory equipment and supplies**  Safety ratings: Low = Biosafety measures are poor, laboratory equipment is lacking or in poor condition, or there are no protective measures; Aver- age = Biosafety measures are in place, the equipment is in fair condition, and some measures provide partial protection; High = Biosafety measures are in place, equipment is in good condition, well-secured and measures provide good protection. |  |  |  |  |
| **156. Condition and safety of medical equipment in emergency care services unit**  Safety ratings: Low = The medical equipment is lacking or in poor condi- tion or there are no protective measures; Average = The equipment is  in fair condition and some measures provide partial protection; High  = Equipment is in good condition, well-secured and measures provide good protection. |  |  |  |  |

Continue >>

| **157. Condition and safety of medical equipment in intensive or interme- diate care unit**  Safety ratings: Low = The medical equipment is lacking or in poor condi- tion, or there are no protective measures; Average = The equipment is  in fair condition and some measures provide partial protection; High = Equipment is in good condition, is well-secured and measures provide good protection. |  |  |  |  |
| --- | --- | --- | --- | --- |
| **158. Condition and safety of equipment and furnishings in the pharmacy** Safety ratings: Low = The equipment in the pharmacy is lacking or in poor condition, or there are no protective measures; Average = The equipment is in fair condition and some measures provide partial protection; High  = Equipment is in good condition, is well-secured and measures provide good protection. |  |  |  |  |
| **159. Condition and safety of equipment and supplies in the sterilization services**  Safety ratings: Low = Equipment is lacking or in poor condition, or there are no protective measures; Average = Equipment is in fair condition and some measures provide partial protection; High = Equipment is in good condition, is well-secured and measures provide good protection. |  |  |  |  |
| **160. Condition and safety of medical equipment for obstetric emergen- cies and neonatal care**  Safety ratings: Low = Equipment is lacking or in poor condition, or there are no protective measures; Average = Equipment is in fair condition and some measures provide partial protection; High = Equipment is in good condition, is well-secured and measures provide good protection. |  |  |  |  |
| **161. Condition and safety of medical equipment and supplies for emer- gency care for burns**  Safety ratings: Low = Equipment is lacking, is in poor condition, or there are no protective measures; Average = Equipment is in fair condition and some measures provide partial protection; High = Equipment is in good condition, is well-secured and measures provide good protection. |  |  |  |  |
| **162. Condition and safety of medical equipment for nuclear medicine and radiation therapy**  Safety ratings: Low = Equipment is lacking, is in poor condition, or there are no protective measures; Average = Equipment is in fair condition and some measures provide partial protection; High = Equipment is in good condition, is well-secured and measures provide good protection.  IF THE HEALTHCARE FACILITIES DOES NOT HAVE THESE SERVICES, LEAVE BOXES BLANK AND PROVIDE COMMENT. |  |  |  |  |
| **163. Condition and safety of medical equipment in other services** Safety ratings: Low = More than 30% of equipment is at risk of material or functional failure and/or equipment puts the entire service’s operation at direct or indirect risk; Average = Between 10% and 30% of equipment is at risk of loss; High = Less than 10% of equipment is at risk of loss. |  |  |  |  |
| **164. Medicines and supplies**  Safety ratings: Low = Nonexistent; Average = Supply covers less than 72 hours at maximum capacity; High = Supply guaranteed for at least 72 hours at maximum healthcare facilities capacity. |  |  |  |  |
| **165. Sterilized instruments and other materials**  Safety ratings: Low = Nonexistent; Average = Supply cover less than 72 hours at maximum capacity; High = Supply is guaranteed for at least 72 hours at maximum healthcare facilities capacity. |  |  |  |  |

Continue >>

| **166. Medical equipment specifically used in emergencies and disasters** Safety ratings: Low = Nonexistent; Average = Supply covers less than 72 hours at maximum healthcare facilities capacity; High = Supply guaranteed for at least 72 hours at maximum healthcare facilities capacity. |  |  |  |  |
| --- | --- | --- | --- | --- |
| **167. Supply of medical gases**  Safety ratings: Low = Less than 10 days’ supply; Average = Supply for between 10 and 15 days; High = Supply for at least 15 days. |  |  |  |  |
| **168. Mechanical volume ventilators**  Safety ratings: Low = Nonexistent; Average = Supply covers less than 72 hours at maximum healthcare facilities capacity; High = Supply guaranteed for at least 72 hours at maximum healthcare facilities capacity. |  |  |  |  |
| **169. Electromedical equipment**  Safety ratings: Low = Nonexistent; Average = Supply covers less than 72 hours at maximum healthcare facilities capacity; High = Supply guaranteed for at least 72 hours at maximum healthcare facilities capacity. |  |  |  |  |
| **170. Life-support equipment**  Safety ratings: Low = Nonexistent; Average = Supply covers less than 72 hours at maximum healthcare facilities capacity; High = Supply guaranteed for at least 72 hours at maximum healthcare facilities capacity. |  |  |  |  |
| **171. Supplies, equipment or crash carts for cardiopulmonary arrest** Safety ratings: Low = Nonexistent; Average = Supplies and equipment for cardiopulmonary emergencies (or crash carts) in good condition but cover less than 72 hours at maximum healthcare facilities capacity; High = Supplies and equipment for cardiopulmonary emergencies (or crash carts) guar- anteed in good condition and adequate supplies for at least 72 hours at maximum healthcare facilities capacity. |  |  |  |  |

Comments on the results of Form 2, Module 3.

................................................................................................................................................................................................................................................................................................................

................................................................................................................................................................................................................................................................................................................

................................................................................................................................................................................................................................................................................................................

................................................................................................................................................................................................................................................................................................................

................................................................................................................................................................................................................................................................................................................

................................................................................................................................................................................................................................................................................................................

................................................................................................................................................................................................................................................................................................................

................................................................................................................................................................................................................................................................................................................

................................................................................................................................................................................................................................................................................................................

................................................................................................................................................................................................................................................................................................................

Name/signature of evaluator(s).........................................................................................................................................................................................................................................

**Module 4:** Structural Safety

| **4.1 Prior events affecting healthcare facilities safety** | **Safety level** | | | **Observations (evaluators’ comments)** |
| --- | --- | --- | --- | --- |
|  | Low | Average | High |  |
| **172. Prior major structural damage or failure of the healthcare facilities building(s)** *Safety ratings: Low = Major damage and no repairs; Average = Moderate damage and building only partially repaired; High = Minor or no damage, or building fully repaired.*  *IF SUCH AN EVENT HAS NOT OCCURRED IN THE VICINITY OF THE HEALTHCARE FACILITIES, LEAVE BOXES BLANK AND PROVIDE COMMENT.* |  |  |  |  |
| **173. Healthcare facilities built and/or repaired using current safety standards**  *Safety ratings: Low = Current safety standards not applied; Average = Current safety standards partially applied; High = Current safety standards fully applied.* |  |  |  |  |
| **174. Effect of remodelling or modification on the structural behaviour of the healthcare facilities**  *Safety ratings: Low = Major remodelling or modifications have been carried out with major compromising effect on the performance of the structure; Av- erage = Moderate remodelling and/or modifications with minor effect on the performance of the structure; High = Minor remodelling and/or modifications; no modifications were carried out; or major remodelling and/or modification enhancing the structural behaviour or having no negative effect.* |  |  |  |  |
| **4.2 Building integrity** | **Safety level** | | | **Observations (evaluators’ comments)** |
|  | Low | Average | High |  |
| **175. Structural system design**  *Safety ratings: Low = Poor structural system design; Average = Moderate structural system design; High = Good structural system design.* |  |  |  |  |
| **176. Condition of the building**  *Safety ratings: Low = Cracks on the ground and first floors; Major deteriora- tion caused by weathering or normal ageing; Average = Some deterioration caused only by weathering or normal ageing; High = No deterioration or cracks observed.* |  |  |  |  |
| **177. Condition of the construction materials**  *Safety ratings: Low = Rust with flaking; cracks larger than 3mm (concrete), excessive deformations (steel and wood); Average = Cracks between 1 and 3 mm present (concrete), moderate and visible deformations (steel and wood) or rust with no flaking; High = Cracks less than 1 mm (concrete), no visible deformations; no rust.* |  |  |  |  |
| **178. Interaction of nonstructural elements with the structure**  *Safety ratings: Low = Partition walls rigidly attached to the structure, sus- pended ceilings or facades interacting with the structures, damage would have significant effect on the structure; Average = Some of the preceding nonstructural elements interacting with the structures, damage would not affect the structure; High = There are no nonstructural elements affecting the structure.* |  |  |  |  |
| **179. Proximity of buildings (for earthquake-induced pounding)**  *Safety ratings: Low = Separation is less than 0.5% of the height of the shorter of two adjacent buildings; Average = Separation is between 0.5% and 1.5% of the height of the shorter of two adjacent buildings; High = Separation is more than 1.5% of the height of the shorter of two adjacent buildings.*  *IF THE HEALTHCARE FACILITIES IS NOT IN A HIGH/MODERATE SEISMIC ZONE, THEN LEAVE BOXES BLANK AND PROVIDE COMMENT.* |  |  |  |  |

Continue >>

| **180. Proximity of buildings (wind tunnel effect and fire)**  *Safety ratings: Low = Separation less than 5 m; Average = Separation between 5 m and 15 m; High = Separation more than 15 m.* |  |  |  |  |
| --- | --- | --- | --- | --- |
| **181. Structural redundancy**  *Safety ratings: Low = Fewer than three lines of resistance in each direction; Average = Three lines of resistance in each direction or lines without orthogo- nal orientation; High = More than three lines of resistance in each orthogonal direction of the building.* |  |  |  |  |
| **182. Structural detailing, including connections**  *Safety ratings: Low = No evidence of engineered building records, or built according to an old design standard; Average = Built according to previous design standards and no retrofitting work to a current standard; High = Built according to a current standard.* |  |  |  |  |
| **183. Ratio of column strength to beam strength**  *Safety ratings: Low = Strength of beams is obviously greater than strength of columns; Average = Strength of beams is similar to strength of columns; High*  *= Strength of columns is greater than strength of beams.* |  |  |  |  |
| **184. Safety of foundations**  *Safety ratings: Low = No evidence that foundations were designed according to standards (foundation size, soil survey) and/or there is evidence of damage; no plans are available; Average = Little evidence (drawings, soil survey) that foundations were designed according to standards; and/or there is evidence for moderate damage; High = Strong evidence that foundations were de- signed according to standards with strong evidence of no damage.* |  |  |  |  |
| **185. Irregularities in building structure plan (rigidity, mass, resistance)** *Safety ratings: Low = Shapes are irregular and structure is not uniform; Aver- age = Shapes on plan are irregular but structure is uniform; High = Shapes on plan are regular and structure has uniform plan, and there are no elements that would cause significant torsion.* |  |  |  |  |
| **186. Irregularities in elevation of buildings**  *Safety ratings: Low = Significant discontinuous or irregular elements, signifi- cant variation in elevation of buildings; Average = Several discontinuous or irregular elements, some variation in the elevation of buildings; High = No significant discontinuous or irregular elements, little or no variation in eleva- tion of buildings.* |  |  |  |  |
| **187. Irregularities in height of storeys**  *Safety ratings: Low = Height of storeys differs by more than 20%; Average = Storeys have similar heights (they differ by less than 20% but more than 5%); High = Storeys are of similar height (they differ by less than 5%).* |  |  |  |  |
| **188. Structural integrity of roofs**  *Safety ratings: Low = Monopitch or flat light roofs, and/or large roof over- hangs; Average = Pre-stressed concrete roof, gable roof with gentle slope, satisfactorily connected, no large roof overhangs; High = Reinforced cast in place on concrete roof deck or hipped light roof, satisfactory connections, no large roof overhangs.* |  |  |  |  |
| **189. Structural resilience to hazards other than earthquakes and strong winds**  *Safety ratings: Low = Low structural resilience to hazards present at the site of the healthcare facilities; Average = Satisfactory structural resilience (taking account of structural risk reduction measures in place); High = Good structural resilience (taking account of risk reduction measures in place).* |  |  |  |  |

Continue >>

Comments on the results of Form 2, Module 2. (Include reference to the building type(s), structural system(s) and age(s) of buildings. Attach site plan, list all buildings and indicate those that were assessed.)

................................................................................................................................................................................................................................................................................................................

................................................................................................................................................................................................................................................................................................................

................................................................................................................................................................................................................................................................................................................

................................................................................................................................................................................................................................................................................................................

................................................................................................................................................................................................................................................................................................................

................................................................................................................................................................................................................................................................................................................

................................................................................................................................................................................................................................................................................................................

................................................................................................................................................................................................................................................................................................................

................................................................................................................................................................................................................................................................................................................

................................................................................................................................................................................................................................................................................................................

Name/signature of evaluator(s).........................................................................................................................................................................................................................................
