# Supplementary material for: Disaster safety assessment of primary healthcare facilities: a cross-sectional study in Kurdistan province of Iran
Source: BMC Emerg Med. 2021 Feb 23;21:23. doi: 10.1186/s12873-021-00417-3 (PMC7903750; doi:10.1186/s12873-021-00417-3)
Supplement: Supplementary file 2 — Additional file 2. [file 12873_2021_417_MOESM2_ESM.pdf]

EDITORIAL CERTIFICATE LETTER

This document is to certify that the manuscript listed below was edited for proper English language, grammar, punctuation, spelling, and overall style by one of the highly qualified subject-expert native English speaking editors at **NativeEnglishEdit.com**

The substantive content of the article mentioned below remains the full responsibility of the author/authors:

TITLE OF ARTICLE:
DISASTER SAFETY ASSESSMENT OF PRIMARY HEALTHCARE FACILITIES: A CROSS-SECTIONAL STUDY IN KURDISTAN PROVINCE OF IRAN

AUTHOR(S):
AREZOO YARI, YADOLAH ZAREZADEH, FARIN FATEMI, ALI ARDALAN, SIAMAK VAHEDI, HOMA YOUSEFI- KHOSHSABEGHE, MOHSEN SOUFI BOUBAKRAN, FARZAM BIDARPOOR, MOHAMAD ESMAEIL MOTLAGH

REFER CODE:
250H ET-2018-33332083-22221771 YARI -2957

Native English Edit
www.NativeEnglishEdit.com

Documents receiving this certification should be English-ready for publication; however, the author has the ability to accept or reject our suggestions and changes.
This certificate may be verified at:
www.NativeEnglishEdit.com
London
East End Road 27, N 3 3QT
United Kingdom
